# Effect of Modified Greenhouse Drying Technology on the Physicochemical Quality of Cameroonian Cocoa Beans

**DOI:** 10.1155/2022/9741120

**Published:** 2022-11-17

**Authors:** Banboye D. Frederick, Ngwa Martin Ngwabie, Nde Divine Bup

**Affiliations:** ^1^Department of Food and Bioresource Technology, College of Technology, University of Bamenda, P.O. Box 39, Bamenda, Cameroon; ^2^Department of Agricultural and Environmental Engineering, College of Technology, University of Bamenda, P.O. Box 39, Bamenda, Cameroon

## Abstract

Several benefits give credence to the importance of maintaining the reference values of the physicochemical properties of cocoa beans at all stages along the cocoa chain. Every drying method confers significant effects on quality with implications on demand and prices. In this work, the modified greenhouse dryers were tested as potential equipment for the production of high quality cocoa beans. To ascertain their validity for use in the cocoa chain, moisture content, pH, free fatty acid, total polyphenol, peroxide value, total phenols, condensed tannins, and flavonoids were investigated. In relation to reference values, all the samples were of good quality. In terms of moisture content, pH, total acidity, free fatty acid, total polyphenol content, phenol content, flavonoids, and peroxide value, the samples fell within the acceptable values. Total phenols, flavonoids, and condensed tannins were within the recommended range. Samples dried in the open sun dryer and modified greenhouse dryer with fleece of cotton presented the highest amounts of total phenols, flavonoids, and condensed tannins. For total fat content, the samples dried in modified greenhouse dryers equipped with fleece of polyester and the conventional greenhouse dryer were distinctly of grade one quality.

## 1. Introduction

In Cameroon, cocoa (*Theobroma cacao L.)* is harvested throughout the year, with peak from November to January [1, 2]. Though flourishing in the humid tropics, the beans must be properly dried after fermentation to prevent spoilage, enhance preservation, and transportation. Secondary products from cocoa beans such as chocolates and beverages constitute the basic cocoa foods in many countries [3], but their quality is largely dependent on postharvest unit operations from the farm to the factory. Due to its high medicinal values, consumption of cocoa products and use in the pharmaceutical industry had increased. In 2021, global production was 5.024 million tonnes, the top five producers being Cote d'Ivoire, Ghana, Indonesia, Nigeria, and Cameroon, having produced 2.034.000, 883.652, 659.776, 328.263, and 295.028 tonnes, respectively, [4]. Africa accounts for 77% of world output, while the Americas, Asia, and Oceania account for 17% and 6%, respectively. The Netherlands is the biggest importer of cocoa powder, purchasing about $220 million USD each year [5].

The economy of Cameroon is greatly agrarian, involving about 80% of the population. Annually, agroproducts contribute about 40% of the Gross Domestic Product, the major export crops being cocoa, coffee, and cotton [6]. These constitute about 90% of the farmers' income, indicating the significant role of agriculture in creation of employment to the rural population [7]. Annual productivity is expected to reach about 650.000 tonnes by 2022, and if linked to quality improvement, will attract high prices at the international markets [8].

Cocoa products are cherished for their rich flavour and increase in metabolic energy [9]. Efforts to correlate the physicochemical properties of cocoa beans to nutritional and health benefits have shown a close association of high polyphenol content to reduction of inflammation and improvement of blood cholesterol levels. Flavonoids lower blood pressure, support neurone production, improve brain function, and prevent age-related brain degeneration [5]. Reduction in risk of heart diseases, diabetes, asthmatic attacks, promotion of healthy skin, teeth, weight loss, and improving heart health have been observed [10]. To ensure this consistency in health, the reference values for the physicochemical properties of cocoa beans at all stages along the cocoa chain should be maintained.

Being a key farm-based unit operation, drying of cocoa beans is carried out based on the indigenous and acquired knowledge of the farmers. While high temperatures and quick drying by heated dryers may stop the biochemical reactions started during fermentation, slow and prolonged drying may lead to contamination by fungi and vermin, loss of quality, and consequent poor prices. Irrespective of how conducive, fast, and cheap the drying method may be, its efficiency and suitability can only be guaranteed if the quality of the dried product meets the expected nutritional, sensory, health, and market standards.

Products dried in the greenhouse dryer are protected from external hazards such as rain, insects, and animals and usually of superior quality compared to those dried in open sun [11]. Greenhouse drying of cocoa beans confer advantages of higher drying temperatures, shorter drying time, less manual labour, and improved quality produce compared to that dried in the open sun [1, 12]. Fleece of various types have been used extensively in greenhouse agriculture for soil solarisation, during which soil temperatures may rise up to 70°C [13]. The degree of rise in soil temperatures has been attributed to the thickness, colour, and specific heat capacity of the material [14]. Although information on the use of greenhouse dryers equipped with fleece is limited in literature, the findings by Banboye et al., [1], indicated that further investigations on the type and thickness of fleece materials could lead to further increase in drying temperatures, reduction in drying time, and improvement in the quality of the dried cocoa beans. In an effort to standardize the drying process and further valorize the Cameroonian cocoa beans, the objective of this work was to investigate the effect of modified greenhouse dryers equipped with black fleeces of polyester, wool, and cotton on the physicochemical quality attributes of the dried cocoa beans. This paper gives the description of the drying process, methods of quality analysis, and the comparative discussion of the quality evaluation of the dried cocoa beans using these dryers. Some of the constituents are evaluated, and whose values affect the suitability of cocoa beans at the international market include moisture content, pH, free fatty acids, total polyphenols, peroxide value, total phenols, condensed tannins, and flavonoids [7].

## 2. Materials and Methods

### 2.1. Construction of the Dryers

**Figure 1:**
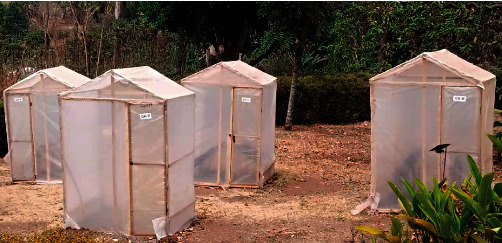
Constructed dryers used in the cocoa bean drying.

**Figure 2:**
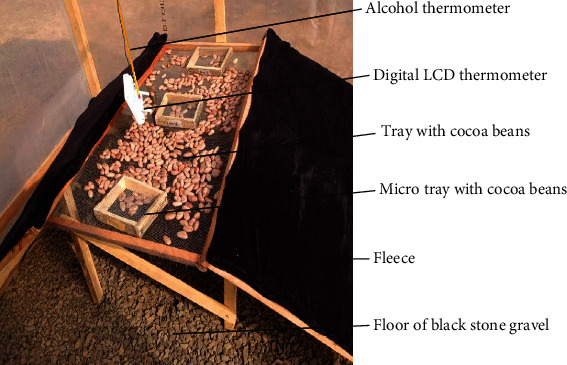
Interior of the modified greenhouse dryer showing the thin layer drying of cocoa beans.

Five dryers of the format described by Banboye et al. [1] were constructed with modifications (type and thickness of fleece) at the University of Bamenda in December 2020. These included two controls—conventional greenhouse (CGHD) and the open sun (OSD) dryers—and three modified greenhouse dryers with black fleeces of polyester (P3X, 7.86 mm), cotton (CTN1X, 2.56 mm), and wool (W1X, 1.56 mm) (Figures 1 and 2).

### 2.2. The Drying Process

A batch of 150 ripe cocoa pods of varied sizes and mixed varieties were obtained from a local farmer, in Makenene, Cameroon (latitude 4.883^o^ N, longitude 10.794°E), and transported to the Food Technology Laboratory of the University of Bamenda (lat. 6.01^o^N, long. 10.26°E) for the drying process. After inspection for defects, pods in good conditions were selected for the research. The basket method of fermentation as described by Neimenak et al. [15] and Banboye et al. [1] was used as follows: after breaking the pods and removing the beans manually using a blunt piece of wood, they were put in a wooden basket lined with fresh banana leaves, covered tightly, and allowed in a closed room for six days, with two turnings after 48 and 96 hours, respectively. The fermentation was completed when the bean colour had changed to brown and temperature risen to 41°C.

The 9 kg of fermented beans were shared into three equal parts (3 kg), sealed in polythene bags, and preserved in a freezer at -5°C for drying in triplicates. At the start of drying for each replicate, the beans were soaked in water overnight (12 hours) to homogenize the moisture content and soften the testa and drained for 15 minutes using a plastic kitchen strainer [16]. Drying proceeded each day from 8 : 00 am to 5 : 00 pm, for three days for all replicates. The beans were considered dry when weight losses were <0.1 g for three consecutive readings. The dried samples were double sealed in transparent polyethene bags and stored in the freezer at -5°C until the analysis. The physicochemical analyses were carried out at the Laboratory of Biophysics, Food Biochemistry and Nutrition of the University of Ngaoundere in Cameroon.

### 2.3. Sample Preparation for Physicochemical Analyses

#### 2.3.1. Production of Cocoa Bean Powder

Each sample was deshelled, winnowed and clean nibs ground into fine, homogeneous powder using an electric blender (Vitamix E310, Explorian Blender, 48 oz), packaged in transparent polyethylene bags, and stored at –5°C for further analyses.

#### 2.3.2. Defatting

Using hexane (95%) as the solvent, fats were removed from the cocoa powder using Soxhlet extraction method for 8 hours. Based on the principle of differential solubilities of oils in extraction solvents, the residual solvent was evaporated and the oil separated. The defatted powder was oven dried at 40°C for 15 minutes to ensure effective evaporation of the solvent. The dried sample was then used directly for the extraction of phenolic compounds and further analysis.

#### 2.3.3. Moisture Content

The moisture content was determined following the method proposed by the AOAC [17], as described by Ismail and Idriss [18] and Parasanna and Shruthi [19]. This principle is based on weight loss of the sample up to constant mass at 105°C for 24 hours. The moisture content (Mc) in percentage (dry weight basis) was determined using Equation (1).

(1) $$\begin{array}{c} \text{Mc}\left(\%\right)=\frac{M_{1}-M_{2}}{M_{1}-M_{0}}\times 100, \end{array}$$

where *M*_*c*_ refers to the moisture content.

*M*_0_ refers to the mass of the empty beaker.

*M*_1_ refers to the mass of the beaker containing the test sample.

*M*_2_ refers to the mass of the beaker and sample after oven drying.

#### 2.3.4. Determination of the pH

The determination of pH was carried out according to the OICCC method no. 9 as described by Pontillon and Cros [20] and measured using a digital pH meter (CyberScan pH 11, Eutech, Singapore).

#### 2.3.5. Total Fat Content and Free Fatty Acid

The total lipid content was evaluated according to the Russian method (Soxhlet extraction) described by Bourely [21]. This principle is based on the differential solubility of lipids in a hot organic solvent (hexane), with the end point determined by discolouration of the contents of the extractor. The total fat (T_f_) content was calculated using Equation (2). The determination of the free fatty acid content was carried out by titration according to the AOAC method 948.22, [17].

(2) $$\begin{array}{c} T_{f}\left(g/100\text{gDM}\right)=\frac{\left(M_{1}-M_{2}\right)x^{2}}{M_{1}-M_{0}}\times 100\times \frac{100}{DM}, \end{array}$$

where *M*_*o*_ refers to the mass of the empty filter paper bag.*M*_1_ refers to the mass of bag containing the test sample before extraction.*M*_2_ refers to the mass of the full bag containing the test sample after extraction of the oil.*D*_*M*_ refers to the dry matter.

#### 2.3.6. Determination Peroxide Value

Using chloroform, acetic acid, potassium iodide, and sodium thiosulphate, the peroxide value was determined using the starch paste as a coloured indicator based on the method described by IUPAC [22]. The peroxide value (PV) was expressed in milliequivalents of active oxygen per kg of material fat using Equation (3).

(3) $$\begin{array}{c} \text{PV}=1000\times N\frac{V_{0}-V_{1}}{M}, \end{array}$$

where: *M* refers to the mass in *g* of the test sample;*V*_*o*_ refers to the volume of thiosulphate solution used for the blank test;*V*_1_ refers to the volume of thiosulfate solution used for the sample; and*N* refers to the exact normality of the sodium thiosulfate solution used.

#### 2.3.7. Determination of Total Polyphenol Content, Phenolic Compounds, and Condensed Tannins

The evaluation of the polyphenols content was carried out following the procedure described by Kim et al. [23] and Wafa et al. [24]. Calibration was done using an aqueous solution of gallic acid (0.2 g/L), and the results are expressed in mg of gallic acid equivalent/g of dry matter. The total flavonoid contents were determined using the calorimetric method described by Dewanto et al. [25]. The quantity of flavonoids per extract was calculated using a standard prepared from a solution of quercetin (0.1 g/L) and the results were expressed in mg equivalent of rutin/g of dried material. The condensed tannins were evaluated according to the spectrophotometry method described by Sun et al. [26].

#### 2.3.8. Statistical Analysis

Expressed as means and standard deviations, Analysis of Variance (ANOVA), and Duncan's multiple rank test were used to compare and rank the means of the various measurements (*p* < 0.05) using the STATGRAPHICS Plus 5.0 software. Graphs and tables were produced using SigmaPlot version 12.5 and MS Excel 2010.

## 3. Results and Discussion

### 3.1. Moisture Content

Cocoa beans were dried from an initial 54.54% to final moisture contents < 6% (Table 1). The international reference value for dried cocoa beans is 5–5.99% for first grade, 6–6.99% for second grade, and 7–8% for third grade [15, 27]. Further drying below 5% would make the shells more brittle, thereby increasing the likelihood of breakages during handling. These results showed that the cocoa beans were ‘over dried,' conferring the advantage of reduced roasting time. This low moisture content however, is not stable as the beans may readily adsorb moisture upon exposure. The short drying period (three days) supports the efficiency of these dryers, as even a shorter period would still give beans with moisture contents within the acceptable range. In terms of moisture content, effective use of these dryers therefore would resolve the request for the standalone grid independent greenhouse dryer employing renewable energy sources expressed by Ganguly and Ghosh [28].

### 3.2. Total Phenol and Flavonoid Contents

Table 1 gives the contents of total phenols, flavonoids, and condensed tannins for the dried cocoa bean samples. The drying process usually initiates major polyphenol oxidizing reactions (catalyzed by polyphenol oxidase) and giving rise to new flavour components, the characteristic brown colour in dry cocoa beans and the resulting chocolate thereof [29]. Cocoa beans with high polyphenol contents are considered to be of high quality by chocolate producers [30]. These polyphenols are directly responsible for the cherished antioxidant properties of its products, with its characteristic bitter taste caused by flavonols [31, 32].

Cocoa beans dried in OSD and CTN1X present the highest amounts of total polyphenols, flavonoids, and condensed tannins, significantly different from those in CGHD, P3X, and W1X indicating that the cotton fleece had a positive influence on the retention of these components during the drying process. Cocoa beans with high flavonoid contents offer beneficial anti-inflammatory effects by protecting body cells from oxidative damage and preventing the development of cardiovascular disease, diabetes, cancer, Alzheimer, and dementia [33]. This therefore supports the use of the CTN1X as a suitable dryer for cocoa beans as it confers the high retention of antioxidant properties in the beans. Considering these health benefits, a significant increase in demand for polyphenol-rich cocoa beans has been reported since consumers are health conscious [31].

The observed range for total polyphenol was 65.37 ± 1.13 to 85.03 ± 0.10 mgGAE/100 g. When compared to reported values from active dryers in Table 2, it can be observed that passive dryers used in this work gave a fairly higher retention of polyphenols. With the drying temperature < 55°C and drying time of 27 hours, enzymatic degradation could be responsible for the high retention observed. Since these dryers were easy to manage, one could assert that they should be vulgarized for large scale use in the drying of cocoa beans, to maintain this higher quality in terms of polyphenol contents.

### 3.3. Condensed Tannins

Condensed tannins (Table 1) refer to organic polymers such as proanthocyanidins, polyflavonoid tannins, catechol-type tannins, pyrocatecollic type tannins, and flavolans usually formed by the condensation of flavans *[*36*]*. Their high contents in the dry beans indicate a low level of oxidation during the drying process, since they would have been depolarized during the process [37]. With upper reference values of 31%, they are attributed to red-brown, dark-brown, brown, and purple colour of cocoa beans and responsible for the astringency mouth feel in dry cocoa beans [38]. Since high tannin contents in seeds helps in maintenance of dormancy and increase its bactericidal and allopathic properties [39], these concentrations will therefore help in the preventing germination and deterioration of cocoa beans during storage and transportation.

### 3.4. Total Fat Content

The total fat content of the dried cocoa beans ranges from 49.79% (W1X) to 57.17% (CTN1X) (Table 3), indicating that they fall within the reference range of 50-57%. These usually comprise of about 33% oleic acid, 25% palmitic acid, and 33% stearic acid [30, 40–42]. This shows that in terms of fat content, the cocoa beans dried in P3X and CGHD were of grade one quality.

### 3.5. Effect of Drying Methods on pH

Table 3 gives the observed pH values for the dried cocoa beans. The pH of well fermented cocoa beans should range from of 4.5 to 6.5 [16, 43]. During drying, there is outward migration of volatile acids as well as biochemical oxidation of acetic acid from the beans leading to an increase in the alkalinity [44]. Contrary to slow drying which would result in low acidity, poorer colour, and presence of moulds, very rapid drying tends to retain an excessive amount of acids such as acetic acid, which is deleterious to the flavour [29, 45].

Although cocoa beans dried in W1X gave the highest pH (6.62), all the samples have pH above 6.33 indicating that they were mildly acidic. Soaking fermented cocoa beans in water for 90 minutes before drying reduces acidity, increases browning, and improves chocolate flavour [46]. Since the fermented beans were soaked in water overnight before drying, this could have enhanced the outward migration of bean acids during the drying process. These results differ with those of Banboye et al., [1], who observed the pH of 4.65 for the cocoa beans dried in a similar dryer without prior soaking. When the cocoa pods were stored for ten days before fermentation, the pH value of 6.00 was observed, thus giving credence to this work [47]. Also, pH values ranging from 5.39 to 6.71 have been reported in marketable cocoa beans from the south and centre regions of Cameroon [15].

### 3.6. Free Fatty Acid Content

The observed FFA content ranges from 0.96% (P3X) to 2.15% (W1X) as shown in Table 3. Reference values in high quality cocoa beans range from 1 to 1.75% [40, 48]. Dry cocoa beans from P3X, CTN1X, CGHD, and OSD contained acceptable levels of FFA indicating that they were of high quality. Those from W1X gave a higher FFA content (2.15%), which is above the reference value (1.75%) for high grade cocoa beans. Mould growth, moisture content > 6.5%, poor storage, diseased pods, slow drying rate, and action of lipases have been attributed the responsibility for higher FFA bean contents [40]. Since all the samples were from a single batch and given same treatment, further investigations are required to ascertain the cause of the disparity. High FFA content in cocoa beans increase the hardness of cocoa butter, crystallisation properties, and make poor quality chocolate in terms of bloom, tempering, and flavour [49].

### 3.7. The Peroxide Value (PV)

Table 3 gives the observed PVs for the cocoa butter extracted from the cocoa beans samples. It ranges from 2.41 ± 0.05 meq/kg (P3X) to 4.43 ± 0.02 meq/kg (OSD), which are significantly different. While the PV for OSD and W1X are highest, those for CTN1X, CGHD, and P3X are lower. These values fall below the reference value for unprocessed fatty products of 10 meq/kg [50, 51]. Increase in the peroxide value depicts higher degree of rancidity of the unsaturated fats in the cocoa butter contained in the beans. Rancid oils contribute to harmful free radicals in humans that might be responsible for illnesses such as diabetes, alzheimer, and digestive distress [33, 52].

Similar works gave higher PVs values ranging from 7.67 ± 0.30 to 12.41 ± 0.53 meq/kg [51], and equally lower PV values ranging from 1.3 ± 0.05 to 2.3 ± 0.07 meq/kg [53]. In both cases, the cocoa beans were described as less risky for human health if a further processing unit operation (roasting) would be done at high temperatures of 135°C to 155°C. Such would reduce the PV to the recommended 1.0 to 2.0 meq/kg before storage, as is the set standard for all purified oils [51, 52].

## 4. Conclusion

A batch of fermented cocoa beans were dried in OSD, CGHD, P3X, W1X, and CTN1X for three days. The effect of the drying methods on moisture content, pH, free fatty acid, total polyphenol, total phenols, condensed tannins, flavonoids, total fat content, and the peroxide value were analyzed. In terms of moisture content, pH, total acidity, free fatty acid, and total polyphenol content, all the samples were of grade one quality. The phenol, flavonoids, total fat content, and peroxide values for all bean samples were within the reference values for grade one and two bean qualities. The free fatty acid content and the high pH indicate that the beans were of a higher economic value. With respect to bean quality, the dryers were therefore ranked in decreasing order as CNT1X, CGHD, P3X, W1X, and OSD. The use of cotton as fleece material has therefore, led to the production of cocoa beans with better quality attributes that meet the first grade in line with international standards. Further investigations would be required to ascertain the quality effects of these dryers if the cocoa beans were dried during the rainy season and in thicker layers.

## Figures and Tables

**Table 1:** Moisture content and phenolic compounds of the dried cocoa bean samples.

Sample	Drying temp range (°C)	Moisture content (%)	Total phenols (mgGAE/100 gD_w_)	Flavonoides (mg QE/100 gD_w_)	Condensed tanins (mgCE/100 gD_w_)
CGHD	20.0–43.3	4.2 ± 0.17^b^	75.41 ± 0.50^b^	48.84 ± 2.45^b^	17.63 ± 0.74^c^
CTN1X	27.3–50.8	3.2 ± 0.48^b^	85.03 ± 0.10^a^	54.11 ± 0.99^a^	21.52 ± 0.06^a^
OSD	18.1–40.6	5.09 ± 0.03^a^	82.76 ± 1.50^a^	51.69 ± 0.96^a^	21.90 ± 0.20^a^
P3X	25.3–44.1	3.85 ± 0.83^b^	65.37 ± 1.13^c^	47.82 ± 0.36^c^	18.79 ± 1.13^b^
W1X	26.1–49.4	3.37 ± 0.32^b^	72.83 ± 2.30^b^	48.23 ± 0.93^b^	21.65 ± 0.12^a^

CGHD: conventional greenhouse dryer; CTN1X, P3X, and W1X: modified greenhouse dryers with fleece of cotton, polyester, and wool, respectively, and OSD: open sun dryer. GAE: gallic acid equivalent; QE: quercetin equivalent; CE: catechin equivalent; and D_w_: dry weight basis. Superscripts of same letters within same parameter are not significantly different, *p* < 0.05.

**Table 2:** Polyphenol content of cocoa beans dried in some active dryers.

Drying method	Temp/°C	Total polyphenol contents/mgGAE/100 g				References
		40 hrs	32 hrs	24 hrs	12 hrs	
Electric oven	60	51.7 ± 0.6	55.1 ± 1.1	59.4 ± 1.2	64.3 ± 0.3	[31]
	70	56.3 ± 0.3	57.3 ± 0.4	62.6 ± 0.9	67.1 ± 0.6	
	80	34.7 ± 0.9	44.3 ± 0.6	48.2 ± 0.9	62.6 ± 0.3	
Heat pump	28.2	73.9	Drying time undefined			[34]
Hot air	Undefined	45 ± 74				[35]
CGHD	Varied	75.4 ± 0.5	Drying time, 27 hrs			This work
CTN1X		85.0 ± 0.1				
OSD		82.8 ± 1.5				
P3X		65.4 ± 1.1				
W1X		72.8 ± 2.3				

CGHD: conventional greenhouse dryer; CTN1X, P3X, and W1X: modified greenhouse dryers with fleece of cotton, polyester, and wool, respectively, and OSD: open sun dryer. GAE: gallic acid equivalent; superscripts of same letters within same parameter are not significantly different, *p* < 0.05.

**Table 3:** Physicochemical features of the dried cocoa bean samples from the five dryers.

Sample	Total fat (g/100 g FM)	pH	FFA (%)	Acid value (mgKOH/g)	PV (mEq/kg)
CGHD	55.41 ± 0.06^c^	6.43 ± 0.01^b^	1.02 ± 0.02^a^	2.02 ± 0.04^a^	3.03 ± 0.25^c^
CTN1X	57.17 ± 1.03^d^	6.58 ± 0.01^c^	1.46 ± 0.01^b^	2.91 ± 0.01^b^	2.65 ± 0.01^b^
OSD	53.97 ± 0.06^b^	6.46 ± 0.01^b^	1.49 ± 0.03^b^	2.96 ± 0.41^b^	4.43 ± 0.02^d^
P3X	55.35 ± 0.74^c^	6.33 ± 0.04^a^	0.96 ± 0.01^a^	1.91 ± 0.01^a^	2.41 ± 0.05^a^
W1X	49.79 ± 0.29^a^	6.62 ± 0.02^d^	2.15 ± 0.08^c^	4.28 ± 0.28^c^	4.36 ± 0.03^d^

meq/kgFM: milliequivalent/kg of fatty materials; FFA: free fatty acids; PV: peroxide value; CGHD: conventional greenhouse dryer; CTN1X, P3X, and W1X: modified greenhouse dryers with fleece of cotton, polyester, and wool, respectively; and OSD: open sun dryer. The values in specific columns with the same letter were not significantly different, *p* < 0.05.

## Data Availability

The data that support the findings of this study are available on request from the corresponding author. The data are not publicly available due to privacy or ethical restrictions.

## Supplementary Materials

Supplementary Materials Freshly fermented cocoa beans dried in five solar dryers, namely, the open sun (OSD), the conventional greenhouse dryer (CGHD), and the three greenhouse dryers modified with black fleeces of polyester, wool, and cotton materials. The sole source of energy is the sun. The drying air properties were affected by the greenhouse effect and the heat conduction properties of the fleece materials. The following physicochemical quality properties of the dried cocoa beans were analyzed, namely, moisture content, pH, Free fatty acid content, titratable acidity, total fat content, total polyphenols, flavonoid content, condensed tannins, and peroxide value.
