# Supplementary material for: Effect of Modified Greenhouse Drying Technology on the Physicochemical Quality of Cameroonian Cocoa Beans
Source: Int J Food Sci. 2022 Nov 17;2022:9741120. doi: 10.1155/2022/9741120 (PMC9691310; doi:10.1155/2022/9741120)
Supplement: Supplementary Materials — Freshly fermented cocoa beans dried in five solar dryers, namely, the open sun (OSD), the conventional greenhouse dryer (CGHD), and the three greenhouse dryers modified with black fleeces of polyester, wool, and cotton materials. The sole source of energy is the sun. The drying air properties were affected by the greenhouse effect and the heat conduction properties of the fleece materials. The following physicochemical quality properties of the dried cocoa beans were analyzed, namely, moisture content, pH, Free fatty acid content, titratable acidity, total fat content, total polyphenols, flavonoid content, condensed tannins, and peroxide value. [file 9741120.f1.docx]

**Graphical Abstract**


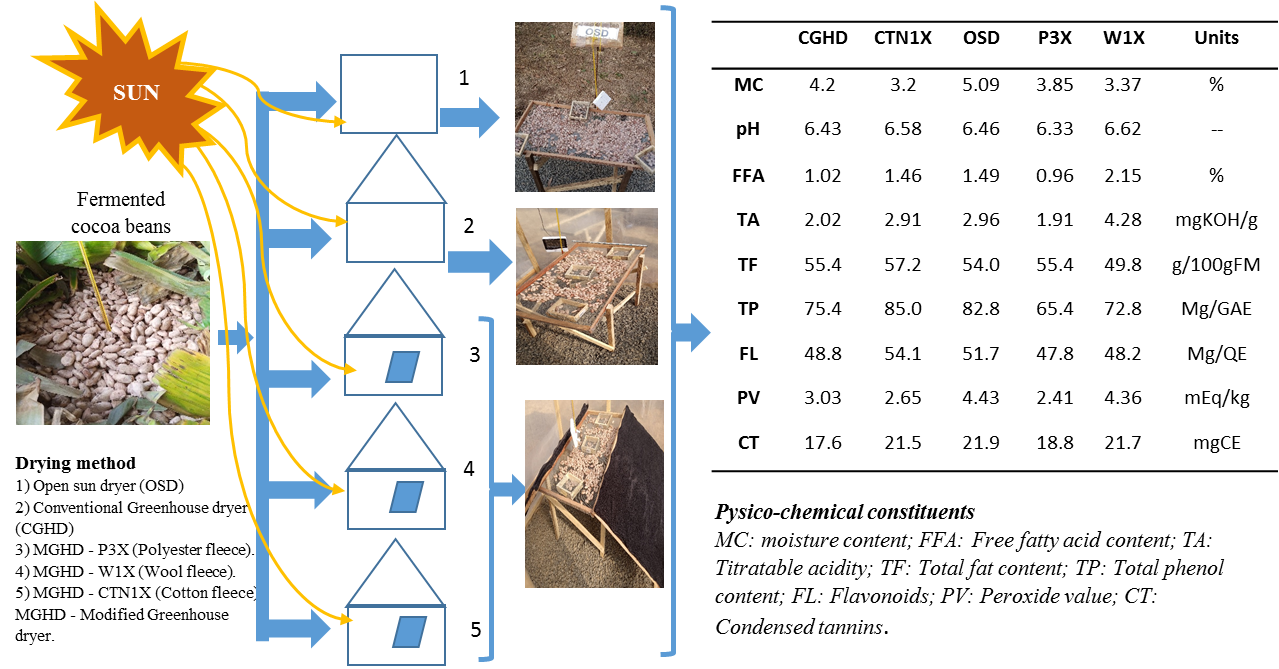


*Effect of Modified greenhouse drying technology on the Physico-chemical quality of Cameroonian cocoa beans*
